# DOE for the formation of the effect of switching between two images when an element is turned by 180 degrees

**DOI:** 10.1038/s41598-020-67590-6

**Published:** 2020-06-30

**Authors:** Anton Goncharsky, Svyatoslav Durlevich

**Affiliations:** Research Computer Center, M.V. Lomonosov Moscow State University, Leninskiye Gory, 1, Building 4, Moscow, 119991 Russia

**Keywords:** Applied mathematics, Displays

## Abstract

An optical security element forming different 2D images when it is turned by 180 degrees is developed and manufactured for the first time. A synthesis technology is developed that incorporates the computation of the beam pattern in elementary hogels with sizes smaller than 100 microns, computation of the phase function of the diffractive optical element (DOE), and formation of the microrelief of the DOE using electron-beam technology. The DOE employed is a multilevel kinoform with an asymmetrical microrelief shaped with a precision of 10 nm. The resulting security feature is easy to control visually, and the DOE is securely protected against counterfeiting. These DOEs are easy to replicate using standard technologies in the manufacturing of embossed holograms and can be used to protect bank notes, securities, and documents against counterfeiting.

## Introduction

The designed diffractive optical element (DOE) is a computer-generated hologram created using advances in the extensively developing field of optics. Computer-generated holograms can form both 2D^1–3^ and 3D^4–9^ images. Optical elements are currently widely used to protect bank notes and documents. The task of prime importance is to develop visual security features. Optical security elements used for visual control are supposed to meet the following standard requirements^1,10^:

- Visual features must be easy to identify and control.
- The technology of synthesis must be knowledge intensive, and the equipment should not be easily available. The DOE must be securely protected against counterfeiting.
- The technology of the production of optical elements must allow mass replication to reduce costs.

In this paper, we discuss the synthesis of relief optical security elements. The synthesis technology includes both the computation of the phase function of the DOE and the formation of the microrelief proper. The technology of the mass production of security DOEs can be subdivided into (1) manufacturing of the original and (2) its mass replication in quantities that can amount to several hundred million. There are two main technologies for manufacturing the originals of security-relief DOEs. The first technology is based on the use of optical radiation to record the originals^1,11^. The second technology is based on the use of electron-beam lithography (EBL). It is interesting that both technologies were first used in projects aimed at protecting bank notes and plastic cards at the same time—in the late 1980s. In 1988, plastic 10-Australian-dollar bank notes were introduced. The original hologram was produced by the Australian company CSIRO using EBL^12^. The security element consisted of binary diffractive gratings. In the same year, Kurz issued bank notes of 5,000 Austrian schillings. The original optical security element also consisted of binary diffractive gratings and was recorded optically^10^.

Currently, hundreds of companies are developing new security elements and producing them en masse. Almost all companies use optical radiation to record the originals of security optical elements. An example of this widespread technology for the synthesis of originals is dot-matrix technology^13^. Only a few companies working in the field of security technologies can afford using EBL to record the originals because e-beam technology is knowledge intensive. The equipment costs dozens of times more than the equipment for optical recording. It could be concluded from this that optical methods for hologram origination are more promising. However, this is not so because in the case of security technologies, their wide use and availability are a drawback rather than an advantage. EBL allows synthesizing originals of security elements that are, in principle, impossible to counterfeit using optical methods of recording. The new security element, which forms different images when turned by 180 degrees, belongs to this class.

EBL outperforms optical methods of microrelief recording in terms of its capabilities. The resolution of e-beam lithographs is several times higher than that achievable with optical methods of recording. However, this is not very important. The main difference is the possibility of forming an asymmetric microrelief with high accuracy. This possibility is used to synthesize the elements considered in this article.

The number of publications where EBL is used to create optical elements has increased in recent years^9, 14,15^. One of the closest studies is our earlier publication^2^. The security feature then proposed is easy to control visually. When the hologram is in the normal position, the observer sees a multicolour image. When the hologram is turned by 180°, the image loses its colour, and the observer sees only a grey silhouette. The main differences between this study and^2^ are as follows. In^2^, the image changes its colour but preserves its 2D geometry. In this study, the geometry of the 2D image changes when the element is turned by 180 degrees. The methods for computing the hogel beam patterns also differ fundamentally in the two cases. In our earlier paper^2^, we used only three different beam patterns, whereas the number of different beam patterns in this study amounts to several hundred. The phase function is computed from the given beam pattern using a well-known iterative algorithm ^16–18^.

The microrelief of an original is synthesized using e-beam technology. E-beam lithographs provide high resolution, which can be as good as 0.01 microns^19–21^. E-beam technology also makes it possible to shape the microrelief with a precision of 10 nm in depth. EBL equipment is used in the stage of the production of the hologram master shim for the security element. The mass replication of these security elements is possible with the standard equipment used for the production of embossed holograms, resulting in the low cost of the final product.

The technology of mass production ensures protection of the microrelief against direct copying. The relief of the security element is covered by one or several layers that are impossible to remove without damaging the microrelief^1^. The DOE developed can be used to protect bank notes, passports, IDs, excise stamps, and brands against counterfeit.

## Results

### Formulation of inverse problems of the synthesis of DOEs and methods for solving them

#### Formulation of the problem

**Figure 1:**
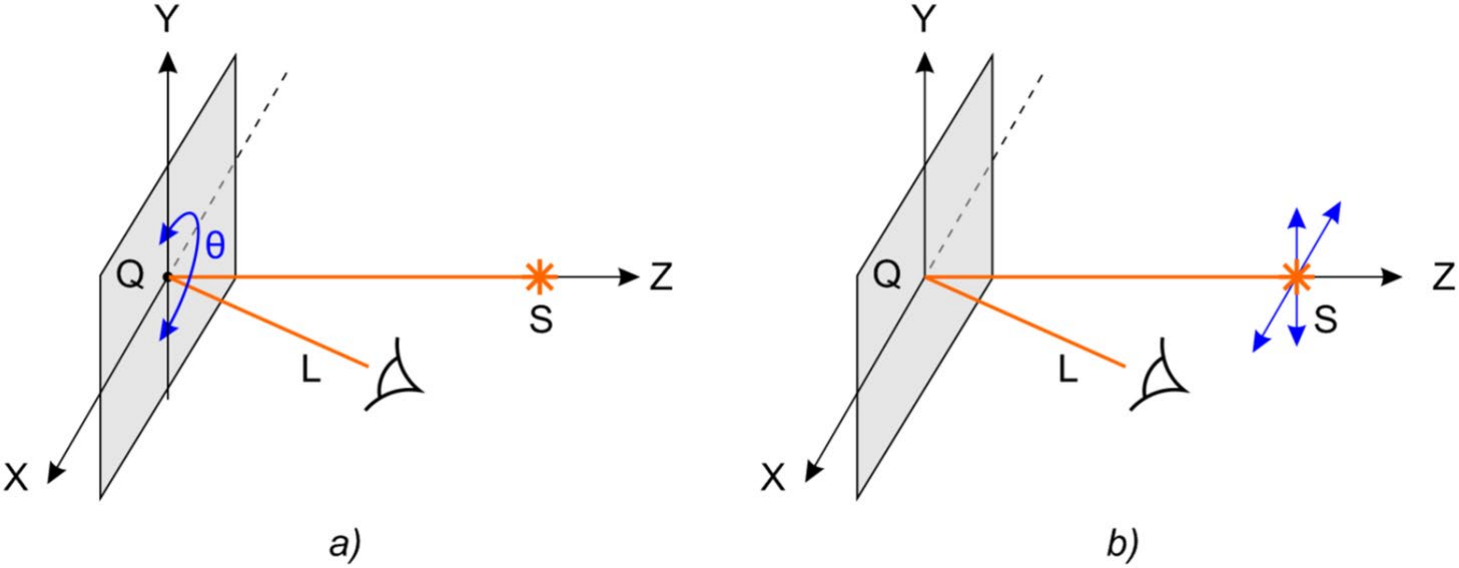
The observation configuration for the visual effect seen is as follows: (**a**) when the DOE is turned by 180 degrees and (**b**) when the source of light is shifted.

Optical security elements have the form of a flat diffraction element whose microrelief forms an image seen by the observer when illuminated by white light. Figure 1a schematically shows the observation of the visual effect of the switching of two 2D images when the DOE is turned by 180 degrees about the Oz axis. Light from white-light source S is incident onto optical element Q. The observer sees the light diffracted from element Q towards direction L. The main security feature is the switching of the image when the optical element is turned by an angle θ equal to 180 degrees. In the case of the normal position of the optical element (θ = 0), the observer sees the image in the plus first order of diffraction, and when the optical element is turned by an angle θ = 180 degrees, the observer sees the image in the minus first order of diffraction. The security feature is easy to control visually—images in the first and minus first orders must differ. At a fixed angle θ in the vicinity of 0 or 180 degrees, the observer may see an extra visual effect. When the source of light is shifted up/down or left/right, the observer sees the kinematic effect of the motion of the image fragments. Figure 1b shows the observation configuration.

**Figure 2:**
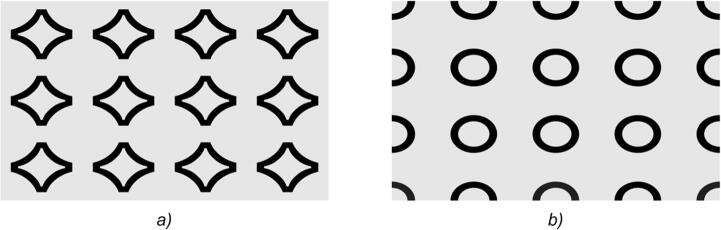
Drawing of the desired image seen at θ = 0° (**a**) and θ = 180° (**b**).

Note that if the microrelief of the DOE is symmetrical, then the images in the plus first and minus first orders always coincide. For the images in the plus first and minus first orders to differ, the DOE must have an asymmetrical microrelief. Figure 2 shows two images seen by the observer when the DOE is turned by 0° and 180°. For the sake of simplicity, we use two binary 2D images. The main visually controlled feature is that the images shown in Fig. 2a, b differ from each other.

**Figure 3:**
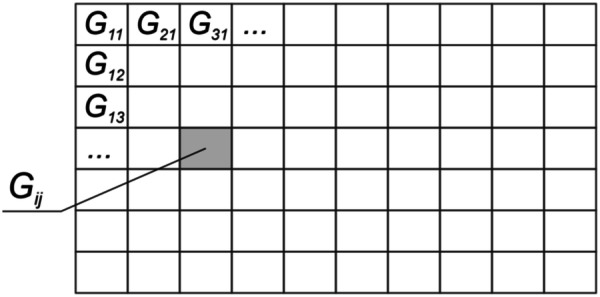
Scheme of the partition of the DOE into hogels.

An important task in the computer synthesis of DOEs is the computation of the phase function *φ*(*ξ*, *η*). It is addressed in two stages. Let us subdivide domain G of the optical element into elementary areas (hogels) G_*ij*_, i = 1… I, j = 1… J (see Fig. 3). The hogel size does not exceed 100 microns, which is beyond the resolution of the human eye. The task of the first stage is to form the beam pattern of each elementary hogel G_*ij*_.

#### Formation of beam patterns

**Figure 4:**
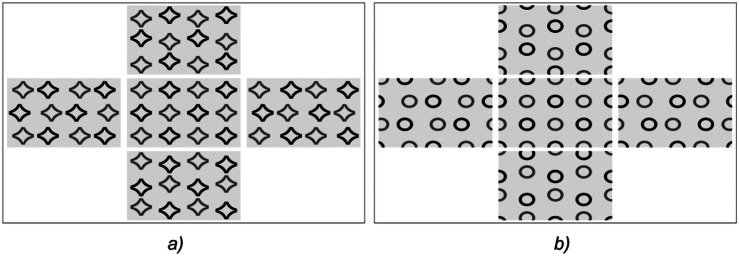
Examples of frames in the vicinity of θ = 0° (**a**) and θ = 180° (**b**).

The beam pattern of a hogel is uniquely determined by image frames K_n,m_, n = 1… N, m = 1… M. Let us denote the image frames in the vicinity of the plus first and minus first order of diffraction as ${\text{K}}^{{({1})}}_{{{\text{n}},{\text{m}}}}$ and ${\text{K}}^{{( - {1})}}_{{{\text{n}},{\text{m}}}}$, respectively. The images shown in Fig. 2 correspond to frames ${\text{K}}^{{({1})}}_{{{\text{N}}/{2},{\text{M}}/{2}}}$ and ${\text{K}}^{{( - {1})}}_{{{\text{N}}/{2},{\text{M}}/{2}}}$, respectively. The number of frames amounts to several hundred. Figure 4a shows five frames in the vicinity of angle θ = 0 when the source of light is shifted left/right and up/down. Figure 4b shows five frames in the vicinity of θ = 180 degrees when the source of light is shifted left/right or up/down.

The proposed variant of the arrangement and displacement of frames forms the so-called parallactic motion, where image fragments shift in the same direction when the optical element is tilted left/right or up/down. Sets of frames ${\text{K}}^{{\left( {1} \right)}}_{{{\text{n}},{\text{m}}}}$ and ${\text{K}}^{{( - {1})}}_{{{\text{n}},{\text{m}}}}$ can also form other dynamic features seen by the observer when the position of the source changes, e.g., image fragments in frames can be rotated rather than shifted.

**Figure 5:**
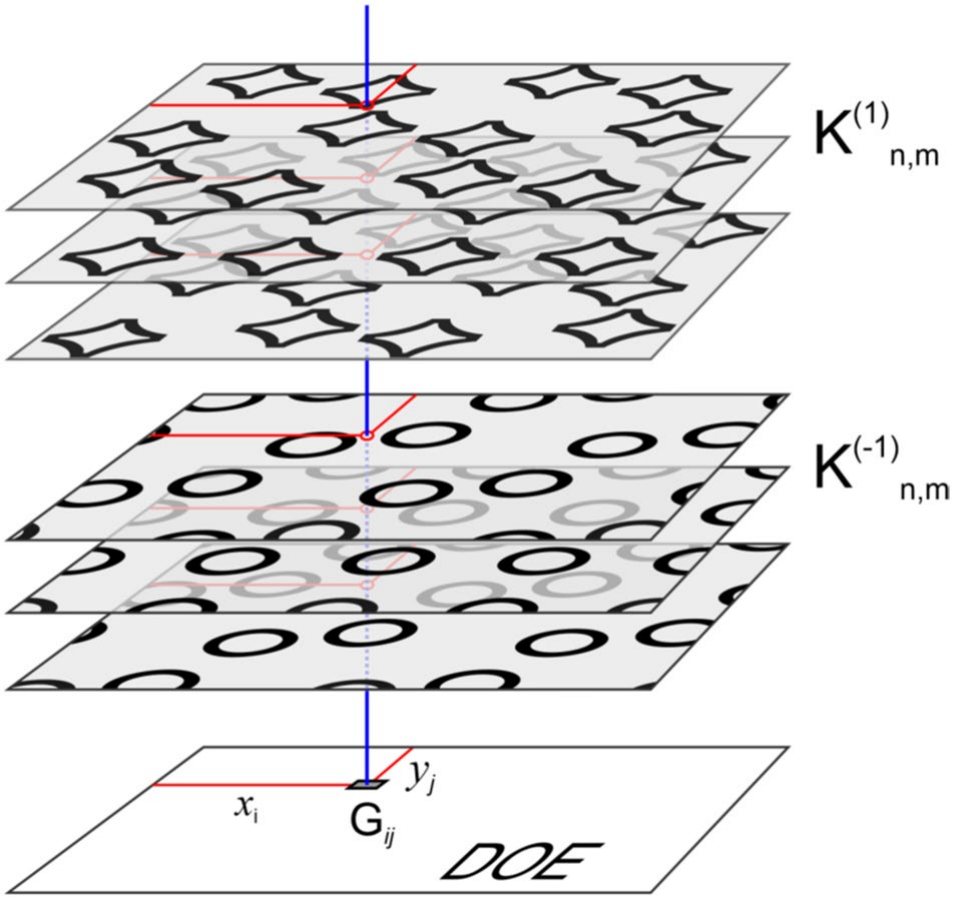
Scheme of the formation of the beam pattern of an arbitrary hogel.

One can use a set of frames in the vicinity of θ = 0 and 180 degrees to compute the beam pattern of each hogel. It is important for the resulting beam pattern of each hogel to bear information about the images seen in both the + 1 and − 1 diffraction orders simultaneously. Figure 5 shows the scheme of the formation of the beam pattern for elementary hogel G_*ij*_.

Let us place the stack of frames ${\text{K}}^{{\left( {1} \right)}}_{{{\text{n}},{\text{m}}}}$ and ${\text{K}}^{{( - {1})}}_{{{\text{n}},{\text{m}}}}$ above the optical element. We then raise the perpendicular from the centre of the hogel. Let’s denote the point of intersection of the perpendicular with frame ${\text{K}}^{{\left( {1} \right)}}_{{{\text{n}},{\text{m}}}}$ and ${\text{K}}^{{( - {1})}}_{{{\text{n}},{\text{m}}}}$ as ${\text{K}}^{{\left( {1} \right)}}_{{{\text{n}},{\text{m}}}} \left( {i, \, j} \right)$ and ${\text{K}}^{{\left( { - {1}} \right)}}_{{{\text{n}},{\text{m}}}} \left( {i, \, j} \right)$, respectively. The intersection point is actually a pixel of the corresponding frame. It can be either black or white.

**Figure 6:**
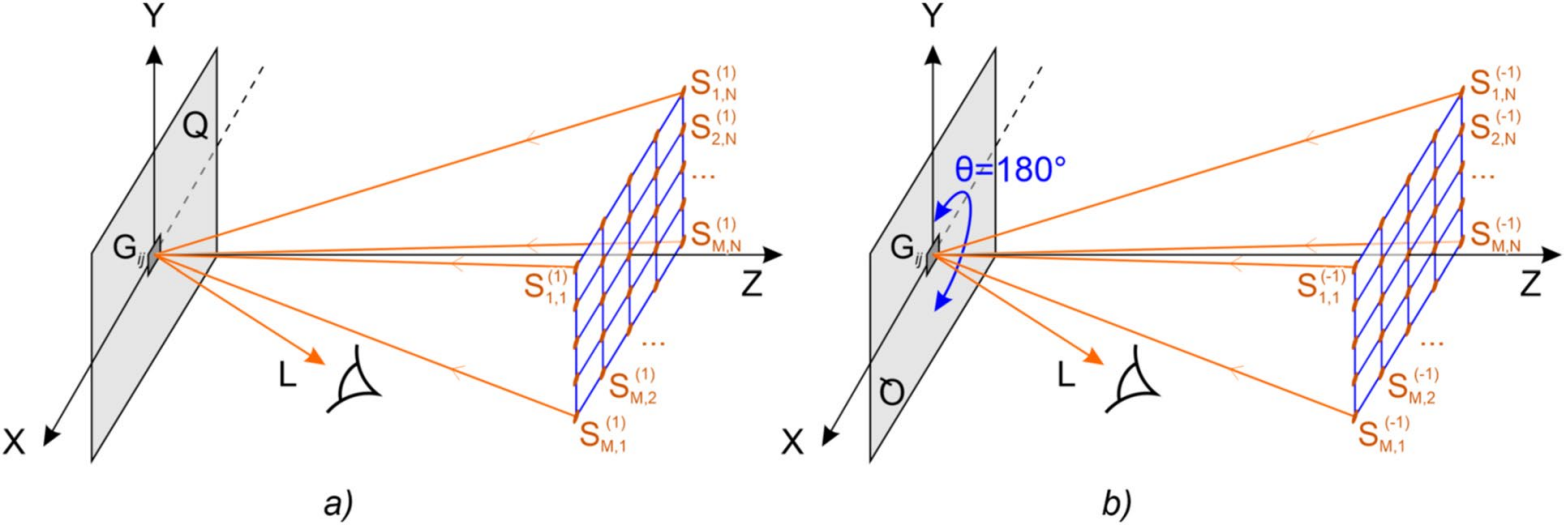
Grid of predefined positions of the source of light S: (**a**) incident light from the source at position ${\text{S}}^{{\left( {1} \right)}}_{{{\text{n}},{\text{m}}}}(i, j)$ is deflected by hogel G_*ij*_ toward observer L and forms the image of pixel ${\text{K}}^{{\left( {1} \right)}}_{{{\text{n}},{\text{m}}}}(i, j)$, (**b**) incident light from the source at position ${\text{S}}^{{\left( -1 \right)}}_{{{\text{n}},{\text{m}}}}(i, j)$ is deflected by hogel G_*ij*_ turned by 180 degrees toward observer L and forms the image of pixel ${\text{K}}^{{\left( {-1} \right)}}_{{{\text{n}},{\text{m}}}}(i, j)$.

**Figure 7:**
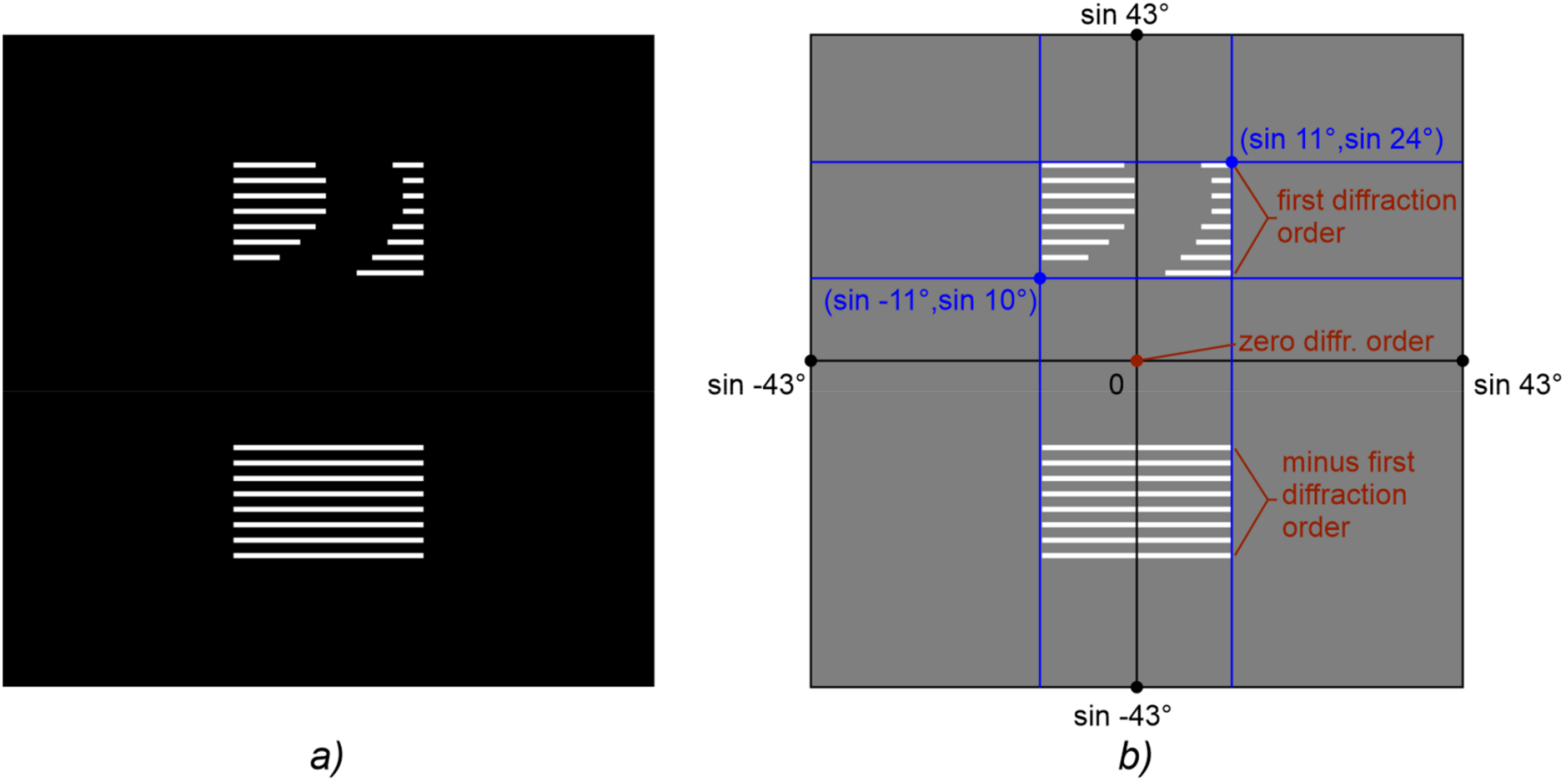
Beam pattern of single hogel G_*ij*_ of the fabricated DOE. The rays deflected to the first and minus first order of diffraction form images of pixels at the position (*i*, *j*) for all frames ${\text{K}}^{{\left( {1} \right)}}_{{{\text{n}},{\text{m}}}}$ and ${\text{K}}^{{( - {1})}}_{{{\text{n}},{\text{m}}}}$, respectively, where m = 1, … M, n = 1,… N (M = 8, N = 50), (**a**) general view, (**b**) the diagram with the diffraction angles shown for the computed wavelength (λ = 546 nm).

If the pixel ${\text{K}}^{{\left( {1} \right)}}_{{{\text{n}},{\text{m}}}} \left( {i, \, j} \right)$ is white, this means that the ray incident from light source S located at the position corresponding to frame K_n,m_ must deviate in the direction towards observer L (Fig. 6). The point (pixel) of the beam pattern that corresponds to the first order of diffraction in this direction is painted white (see Fig. 7). Similarly, for ${\text{K}}^{{\left( { - {1}} \right)}}_{{{\text{n}},{\text{m}}}} \left( {i, \, j} \right)$ the point corresponding to the—1-st order of diffraction is also painted white. The geometry of the optical system is defined to make it easy to compute the ray inclination angles in the spherical coordinate system with the origin coincident with the hogel centre. If the intersecting point is black, then the corresponding ray does not participate in the formation of the image point. If we repeat this procedure for all rays that participate in the formation of all image frames, then we obtain a black-and-white binary image, which determines the beam pattern of hogel G_*ij*_. Figure 7 shows an example of this image. The maximum number of rays in the beam pattern of the hogel coincides with the number of frames.

Our paper^2^, published in 2015, presents an optical security element, which also has the effect of a change in the image when turned by 180 degrees. The new DOE differs fundamentally from that proposed in our earlier study in terms of the visual effects it creates, the method of subdivision into elementary areas, and the method of the computation of the beam pattern of each hogel. In our earlier study^2^, in the case of the normal position of the DOE (in the vicinity of θ = 0 degrees), one could observe a three-colour image consisting of areas with pure spectral colours Red, Green and Blue. When the DOE was turned by θ = 180 degrees, the observer saw the same image but with the areas of pure colours having changed to grey. To implement this effect, three asymmetric beam patterns were formed that corresponded to the three colours of the image. In this paper, unlike our earlier study^2^, the task is to create the effect of the change in images and to have two images with different graphical designs. In addition, the effect of the motion of image elements is implemented. To create these effects, here, for each hogel G_ij_, the beam pattern is computed individually in accordance with the coordinates (*x*_i_, *y*_j_) of the hogel centre (see Fig. 5). The total number of different beam patterns amounts to several hundred.

#### Phase function computation

**Figure 8:**
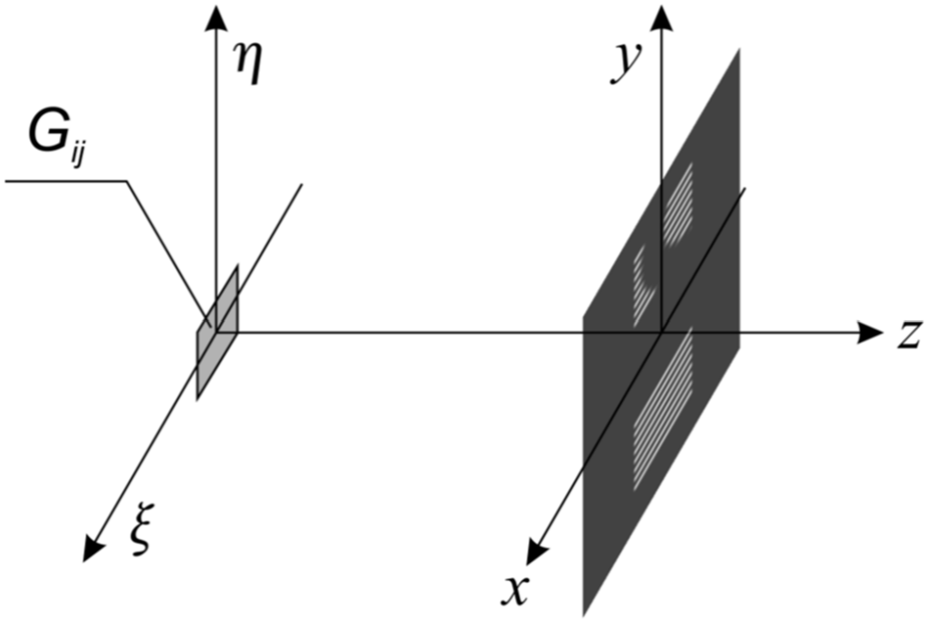
Scheme of the computation of the phase function *φ*(*ξ*, *η*) of hogel G_*ij*_.

In the second stage, the phase function *φ*(*ξ*, *η*) is computed for each elementary hogel G_*ij*_. Figure 8 shows the optical scheme for computing the phase function in hogel G_*ij*_. The hogel is located in the O*ξη* plane of the optical element. In the O*xy* plane, the following beam pattern is defined, which is computed as described above. Let us denote the image as F(*x*, *y*).

The inverse problem consists of reconstructing the phase function *φ*(*ξ*, *η*) in the O*ξη* plane based on the given image F(*x*, *y*) in the *z* = *f* plane. As is well known, F(*x*, *y*) and *φ*(*ξ*, *η*) are related by the following formula^22^:

1 $$\left| {\gamma \iint\limits_{{G_{{ij}} }} {u(\xi ,\eta ,0 - 0)\exp (ik\varphi (\xi ,\eta ))}} \right.\left. {\exp \left\{ {ik\frac{{(x - \xi )^{2} + (y - \eta )^{2} }}{{2f}}} \right\}d\xi d\eta } \right| = \left| {u(x,y,f)} \right| = {\text{F}}(x,y).$$

Here, $u(\xi,\eta,0-0)$ is the scalar wave field of incident light; *u*(*x*, *y*, *f*), the scalar wave field formed by the DOE in the z = *f* plane; *f*, the distance to the observer; *k*, the wave vector; and γ, a known constant.

Equation (1) can be rewritten in operator form as A*φ* = F. Functions *φ* and F belong to the space of quadratically integrable functions. Problem (1) is a standard operator equation of the first kind A*φ* = F. It is an ill-posed problem^23,24^, which can be solved using various methods, e.g., gradient minimization of the functional R(φ) =||A*φ* − F||^2^^25^. One of the first successful iterative methods for solving nonlinear problems (1) was proposed by Lesem et al. more than 50 years ago^18^. This method involves constructing an iterative sequence *φ*_*n*_ minimizing the functional R(*φ*). The algorithm proposed by Lesem et al. has a very useful property of being of a relaxation type^11^: The residual R(*φ*_*n*+1_) of the (*n* + 1)st iteration does not exceed the residual R(*φ*_*n*_) of the *n*th iteration. Lesem’s method can be shown to represent one of the cases of gradient minimization of a functional with a special choice of step at each iteration. In a nutshell, the algorithm can be described as follows.

Let us introduce the following designation:

2 $$w(x,y) = \Phi \{ v\} (x,y) = \frac{k}{2\pi f}\iint\limits_{G} {v(\xi ,\eta ) \cdot \exp \left[ {ik\frac{{(x - \xi )^{2} + (y - \eta )^{2} }}{2f}} \right]d\xi d\eta }.$$

Here, $\Phi \{ v\} (x,y)$ is the Fresnel transform of function *v*. We also use the inverse Fresnel transform $\Phi^{-1}\{w\}$. The iterative process of constructing an approximate solution for the phase function, which is an approximate solution for inverse problem (2), is arranged as follows. Four steps must be performed to complete a single iteration in the iterative algorithm used to solve problem (2). Let *v*^(k)^(*x*, *y*) and *w*^(k)^(*x*, *y*) at the kth iteration be already known. We write functions *v*^(k)^(*x*, *y*) and *w*^(k)^(*x*, *y*) in the form *v*^(k)^(*x*, *y*) = A_0_ exp[*ikφ*_*0*_^(*k*)^(*x*, *y*)] and *w*^(k)^(*x*, *y*) = A_1_ exp[*ikφ*_*1*_^(*k*)^(*x*, *y*)], respectively. Let A_0_(*x*, *y*) be the given amplitude distribution of incident light in the *z* = 0 plane and A_1_(*x*, *y*) be the amplitude distribution in the focal plane *z* = *f* (A_0_ and A_1_ are known real functions). The algorithm for solving the inverse problem consists of the following four consecutive steps:

- *Step 1*
  3 $$\tilde{v}^{(k)} = \Phi \{ v^{(k)} \} (x,y) = W_{k} (x,y)\exp [ik\varphi_{1}^{(k)} (x,y)].$$
- *Step 2*
  4 $$w^{{\left( {\text{k}} \right)}} \left( {x,y} \right) = {\text{A}}_{1} \exp \left[ {ik\varphi_{1}^{(k)} \left( {x,\,y} \right)} \right]\,.$$
- *Step 3*
  5 $$\tilde{w}^{(k)} (x,y) = \Phi^{ - 1} \{ w^{(k)} \} (x,y) = V_{k} (x,y)\exp [ik\varphi_{0}^{(k + 1)} (x,y)].$$
- *Step 4*
  6 $$v^{{\left( {{\text{k}} + 1} \right)}} \left( {x,\,y} \right)\, = {\text{A}}_{0} \left( {x,\,y} \right)\exp \left[ {ik\varphi_{0}^{(k + 1)} \left( {x,\,y} \right)} \right].$$

The combination of *φ*_*0*_^(*n*)^(*x*, *y*) values, i.e., the *n*th iteration of Lesem’s algorithm, is adopted as the approximate solution. A characteristic feature of this iterative method is that it produces a sufficiently good approximate solution after just a few – one or two dozen – iterations with further iterations, resulting in a much slower decrease in the residual functional R(*φ*^(*n*)^). The ill-posedness of inverse problem (1) consists of the fact that iterative sequence *φ*^(*n*)^ may be nonconverging. However, we are addressing the problem of synthesis, where the approximate solution can be considered to be equal to any element of the iterative sequence such that the functional R(*φ*^(*n*)^) does not exceed the given error limit. The phase function *φ*(*ξ*, *η*) is computed by formulas (2–6) at the fixed wavelength corresponding to the maximum sensitivity of the human eye.

**Figure 9:**
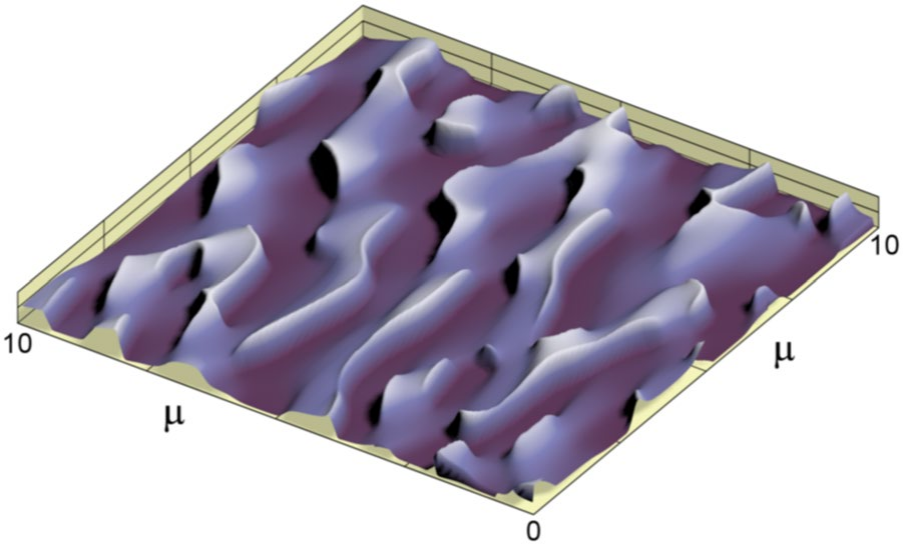
Fragment of the calculated microrelief of a hogel.

As a result of the computations, we obtain the phase function *φ*(*ξ*, *η*) in region G_*ij*_. The phase function for the entire region of the optical element is obtained by performing the corresponding computations for each elementary region. The phase function uniquely defines the microrelief. For example, in the case of normal incidence, the depth of the microrelief is equal to *h*(*ξ*, *η*) = 0.5 *φ*(*ξ*, *η*). Figure 9 shows a fragment of the calculated microrelief of a hogel. As evident from the figure, the microrelief is not symmetric. The microrelief fragment in Fig. 9 has a size of 10 × 10 micron^2^ and a depth of 0.27 microns.

### Production of a DOE sample using e-beam lithography

We use EBL to produce the DOE. This technology provides high resolution, which can be as good as 0.01 microns. The accuracy of the microrelief formation at depth should be on the order of 10 nm. Modern e-beam lithographs open very ample prospects for the formation of fundamentally new optical diffraction elements. In this paper, we discuss diffraction optical elements that can be mass replicated.

Electroforming can be used to produce a nickel shim, which can be utilized for the mass replication of optical elements on an enormous scale, amounting to several billion copies. These optical elements have the same security features as the original, they form the same images as the original, but the cost of mass-replicated products is low. Unfortunately, mass-replication technology imposes certain limitations. Currently, optical elements made using e-beam technology with a resolution of 0.05 microns can be successfully mass replicated. It is this technology that we are addressing in this paper. Optical security elements made by using e-beam technology cannot be imitated using optical methods for the formation of the microrelief. The knowledge-intensive nature of EBL and high cost of the equipment securely protect the optical security elements developed against counterfeiting.

To demonstrate the efficiency of the solutions proposed in this paper, we made a sample diffraction optical element forming the "switch by 180°" security feature. The optical element had a size of 17 × 10.4 mm. The size of the hogel used in the partition of the optical element into elementary areas was 50 microns, and the number of frames used to form the beam pattern of hogels in each of the frame sets ${\text{K}}^{{\left( {1} \right)}}_{{{\text{n}},{\text{m}}}}$ and ${\text{K}}^{{( - {1})}}_{{{\text{n}},{\text{m}}}}$ was equal to 400. The total number of frames was 800. The depth of the microrelief was computed for the fixed wavelength λ = 546 nm. The exposure was performed on a glass plate with a PMMA e-beam resist using a shaped-beam Carl Zeiss ZBA-21 lithography system with a minimal pixel size of 0.1 × 0.1 microns. The microrelief depth of the final DOE produced was 270 nm, and its shaping precision at depth was 10 nm.

**Figure 10:**
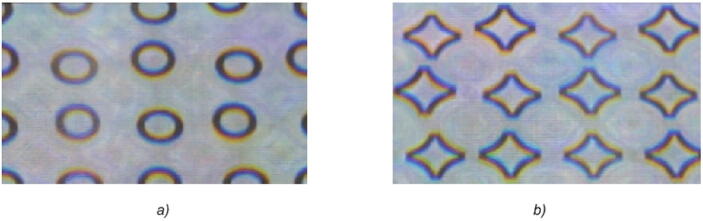
Photos of the produced DOE with an asymmetrical microrelief in different positions: in the vicinity of θ = 0° (**a**) and θ = 180° (**b**). See (Supplementary Video 1).

Figure 10 shows the real photos and video that we obtained in accordance with the observing schemes in Fig. 1a, b, with the DOE illuminated by an extended white-light source. The angular size of the light source was equal to 8 degrees. The video was captured from the nickel master shim of the DOE. As is evident from the video, a turn of the optical element by 180 degrees produced a clear alternation of the two images. When the position of the source of light shifts in the vicinity of 0 and 180 degrees, the observer may see kinematic effects of the motion of the image.

The image quality improves if the DOE is illuminated by monochromatic light. However, given that mobile phone flashlight is the most easily accessible source light, we have to focus on it. The quality of the image in Fig. 10 is nevertheless satisfactory. This effect can be explained as follows. Triangular groove profile has such a feature that diffraction efficiency depends only slightly on wavelength over a wide wavelength range if nonpolarized source of light (e.g., a phone flashlight) is used. In the book^26^ the diffraction efficiency *P* for a triangular-profile blazed grating with the inclination angle close to the average angle of the profile used in this study is estimated to be *P* > 0.5 in the 0.67·λ_0_ < λ < 1.8·λ_0_ wavelength interval, where λ_0_ is the wavelength at which the efficiency reaches its maximum. In our case λ_0_ = 546 nm, and the interval covers the entire optical range and more.

Additionally, for comparison, a DOE was produced by using the same image frames and parameters but with a symmetrical (binary) microrelief with a depth of approximately 130 nm. The microrelief was binarized from the same phase function using the threshold method.

**Figure 11:**
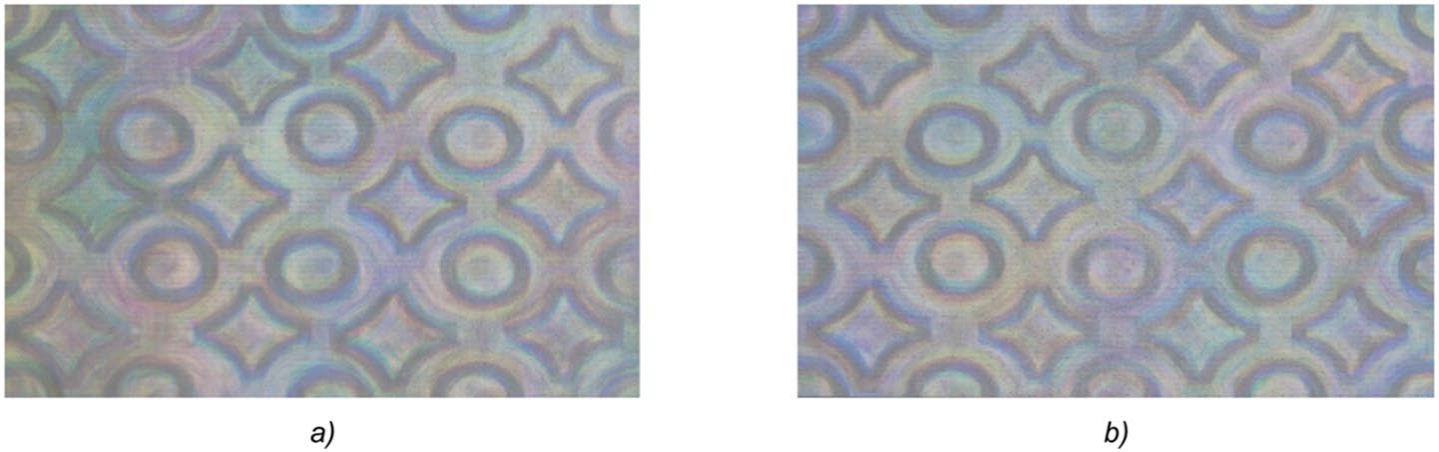
Photos of the produced DOE with a symmetrical (binarized) microrelief in different positions: in the vicinity of θ = 0° (**a**) and θ = 180° (**b**).

As is evident from Fig. 11, the DOE with the symmetrical microrelief provides the same images visible at θ = 0° and θ = 180°, and the images formed are of low contrast. Therefore, the visual effect of the binary DOE is obviously different from the "switch by 180°" visual effect proposed in the paper. The overwhelming majority of the companies synthesizing optical security elements use optical methods of origination. These technologies make it possible to produce only symmetrical microreliefs, and this automatically results in identical images seen at θ = 0° and θ = 180°, as shown in Fig. 11.

## Conclusions

An optical security element forming different images when turned by 180 degrees is developed and made for the first time. The new security feature – switch by 180° – uses the capabilities of EBL. The DOE has the form of a multilevel kinoform with an asymmetrical microrelief. The precision of the microrelief production is 10 nm. The developed security feature can be replicated, which ensures its low cost in the case of mass production. The visual effect is easy to control and is securely protected against counterfeiting. The developed technology can be used to protect bank notes, passports, IDs and brands.

## Methods

The synthesis method developed in the article includes the calculation of the DOE phase function, which has the effect of changing two 2D images when the DOE is rotated through 180 degrees. The synthesis of the DOE includes the calculation of its phase function and the fabrication of the microrelief. The calculation of the phase function is carried out in two stages. In the first stage, the DOE is divided into elementary regions—hogels of 50 microns in size; the total number of hogels for the DOE produced with a size of 20 × 14 mm is 112 000. The radiation pattern of the reflected light is calculated for each hogel. Each hogel is involved in the formation of all K_n_ frames. The total number of frames used is 800. For the calculation, the scheme shown in Fig. 5 is used. The radiation pattern of the reflected light is calculated in the geometrical approach in the finite parametric model. In the second stage, for each elementary hogel, the DOE phase function is calculated according to a given radiation pattern. For the calculation, algorithm (3–6) is used; to increase the calculation speed, a fast Fourier transform is implemented. The total time of calculation of the entire phase function of the DOE is 140 s for radiation patterns in all hogels plus 600 s for the kinoforms in all hogels. All computations are carried out on a PC with AMD Phenom II X6 3.2 GHz CPU and 16 Gb DDR3 memory. The phase function uniquely determines the microrelief of the DOE. The calculations are performed for a fixed wavelength of 546 nm, and the microrelief is formed by using electron beam lithography. A shaped electron beam lithography system is used to form the microrelief, where the minimum beam size is 0.1 × 0.1 microns and the maximum possible beam size is 6.3 × 6.3 microns. The microrelief is formed on a positive PMMA resist with a thickness of 0.5 micron, and the accuracy of the formed microrelief at depth is not greater than 10 nm.

Then, the microrelief is coated with a thin layer of silver using a vacuum evaporation system with a resistive thermal heater. Next, a 0.2 mm-thick nickel master shim is grown in an electroforming bath, and the master shim is used to capture photos and videos for the present article.

## Supplementary information

Supplementary information

Supplementary information
