# Supplementary material for: DOE for the formation of the effect of switching between two images when an element is turned by 180 degrees
Source: Sci Rep. 2020 Jun 30;10:10606. doi: 10.1038/s41598-020-67590-6 (PMC7327009; doi:10.1038/s41598-020-67590-6)
Supplement: Supplementary file 1 — Supplementary information [file 41598_2020_67590_MOESM1_ESM.docx]

Supplementary Information for

**DOE for the formation of the effect of switching between two images when an element is turned by 180 degrees**

**Anton Goncharsky and Svyatoslav Durlevich^*^**

*Research Computer Center, M.V. Lomonosov Moscow State University, Leninskiye Gory, 1, building 4, Moscow 119991, Russia*

**sdurlevich@ya.ru*

**Supplementary Video 1**: Video of the produced DOE with an asymmetrical microrelief. A turn of the optical element by 180 degrees produced a clear alternation of the two images. When the position of the source of light shifts in the vicinity of 0 and 180 degrees, the observer may see kinematic effects of the motion of the image.
